# Post Eclosion Age Predicts the Prevalence of Midgut Trypanosome Infections in *Glossina*

**DOI:** 10.1371/journal.pone.0026984

**Published:** 2011-11-08

**Authors:** Deirdre P. Walshe, Michael J. Lehane, Lee R. Haines

**Affiliations:** Vector Group, Liverpool School of Tropical Medicine, Liverpool, United Kingdom; Federal University of São Paulo, Brazil

## Abstract

The *teneral phenomenon*, as observed in *Glossina* sp., refers to the increased susceptibility of the fly to trypanosome infection when the first bloodmeal taken is trypanosome-infected. In recent years, the term *teneral* has gradually become synonymous with ***unfed***, and thus fails to consider the ***age*** of the newly emerged fly at the time the first bloodmeal is taken. Furthermore, conflicting evidence exists of the effect of the age of the teneral fly post eclosion when it is given the infected first bloodmeal in determining the infection prevalence. This study demonstrates that it is not the feeding history of the fly but rather the age (hours after eclosion of the fly from the puparium) of the fly when it takes the first (infective) bloodmeal that determines the level of fly susceptibility to trypanosome infection. We examine this phenomenon in male and female flies from two distinct tsetse clades (*Glossina morsitans morsitans* and *Glossina palpalis palpalis*) infected with two salivarian trypanosome species, *Trypanosoma (Trypanozoon) brucei brucei* and *Trypanosoma (Nannomonas) congolense* using Fisher's exact test to examine differences in infection rates. Teneral tsetse aged less than 24 hours post-eclosion (h.p.e.) are twice as susceptible to trypanosome infection as flies aged 48 h.p.e. This trend is conserved across sex, vector clade and parasite species. The life cycle stage of the parasite fed to the fly (mammalian versus insect form trypanosomes) does not alter this age-related bias in infection. Reducing the numbers of parasites fed to 48 h.p.e., but not to 24 h.p.e. flies, increases teneral refractoriness. The importance of this phenomenon in disease biology in the field as well as the necessity of employing flies of consistent age in laboratory-based infection studies is discussed.

## Introduction

In the tsetse research community, the term teneral fly has gradually become synonymous with a newly emerged but unfed fly [1]. The word ‘teneral’ is derived from the Latin verb ‘*tener*’, which means young, soft and tender. A teneral tsetse fly is quite easily recognized as it is marked by a soft, ‘soapy-feeling’, exoskeleton and a body coloration that is lighter than that of the mature adults. A teneral fly is still engaged in the process of maturation and has a different physiology and behaviour compared to a fed adult [2]. One of the notable teneral differences is in the susceptibility of the fly to trypanosome infection. Under laboratory conditions, it is firmly established that teneral tsetse flies offered trypanosomes in their first bloodmeal are more susceptible than flies fed trypanosomes in a later bloodmeal [3], [4], [5],[6],[7]. This has become known as the ‘teneral phenomenon’. For this reason, flies for experimental purposes are commonly infected at the first bloodmeal as this maximizes the percentage of infected flies that are available for experiments. There are also reports that the age of the teneral fly post eclosion (p.e.), when it is given this infected first bloodmeal, can be of critical importance in determining the infection prevalence. However, the reports are not consistent. Several authors have found the younger the teneral fly is when given this first infected meal the more susceptible it is [8], [9], [10], [11] but others have not found this to be the case [7], [12], [13]. Although an understanding of the varying susceptibility of teneral flies to infection is crucial to the adequate design of most trypanosome infection experiments in tsetse flies, it is usually not considered in experimental planning. Consequently, we have re-visited this issue and tried to provide more robust data than has previously been available. In addition, we have investigated the phenomenon in two tsetse species (*G. m. morsitans* and *G. p. palpalis*) using two trypanosome species (*T. b. brucei* and *T. congolense*). Also, previously published work has used the unnatural process of using *in vitro* cultured procyclic form (PCF) trypanosomes rather than bloodstream form (BSF) trypanosomes to infect experimental tsetse flies. Thus, we have also investigated the effect of this practice on the teneral phenomenon [14], [15]. We do not seek here to clearly define the mechanisms underpinning this phenomenon, but rather we look to gather the data essential to the robust experimental design of those and other investigations on tsetse-trypanosome interactions.

## Materials and Methods

### Flies and trypanosomes

*Glossina morsitans morsitans* (Westwood), reared in a colony at the Liverpool School of Tropical Medicine (LSTM), were maintained at 26°C with 65–75% relative humidity in a 12 h light, 12 h dark cycle. *Glossina palpalis palpalis* were supplied as puparia from the International Atomic Energy Agency (IAEA) Entomology Laboratories, Siebersdorf, Austria and maintained as flies as described above. Groups of experimental flies were collected over a four hour time period so that “young” tenerals were aged 22 h.p.e.–26 h.p.e. (24 h.p.e. group) and “old” were aged 46 h.p.e.–50 h.p.e. (48 h.p.e. group). For the time course experiment (12 h.p.e.–72 h.p.e.), *G. m. morsitans* male and female flies were collected every 12 hours during a four-hour window (10:00–14:00 and 22:00–02:00) over 3 days. In all experiments, flies were initially sorted by brief chilling at 4°C. These batches of age-grouped flies were then infected with trypanosomes at the first bloodmeal. Subsequently, tsetse were offered a sterile defibrinated horse bloodmeal through silicone membranes every 48 hours [16].

Several lots of murine stabilates of *T. b. brucei* TSW196 [17] and *T. congolense* 1/148 BSF [18] were used for infections. This was to ensure that the observed infection rates were not biased by the ratio of short stumpy forms (*T. b. brucei* BSF) present in the blood at the time of donor exsanguinations [19]. Each infected rodent stabilate was diluted in sterile defibrinated horse blood (TCS Biosciences Ltd., Buckingham, UK) to give a concentration of approximately 5×10^5^ parasites/mL. The volume of blood consumed by tsetse fluctuated between 17–35 µL. This ingested volume translates into a dose of approximately 8.0–17.5×10^3^ trypanosomes per fly. PCF of *T. b. brucei* TSW196 were *in vitro* transformed from BSF at 27°C [20] and subsequently adapted to MEM/10% FBS as described [21], [22]. PCF were harvested in log-phase growth (7×10^5^ trypanosomes/mL), washed twice (800×g) in sterile PBS to remove all traces of medium-derived antibiotics (Pen-Strep) to avoid harming the tsetse flies' enteric symbionts. The PCF were counted using a Neubauer haemocytometer and adjusted to 2.5×10^6^ parasites in 200 µL of PBS before adding to 4.8 mL of defibrinated horse blood (final concentration 5×10^5^ trypanosomes/mL). Experimental flies were first offered an infective bloodmeal for 10 minutes, but were allowed to feed for a further 15 minutes if a large percentage of flies still remained unfed. Unfed flies were manually removed from the cages 24 hours after feeding by chilling flies for 10 minutes at 4°C. Flies were offered a sterile defibrinated horse bloodmeal through silicone membranes 24 hours later, and every 48 hours thereafter [16]. Flies were starved for 48–60 hours prior to dissection 6–8 days after the infectious bloodmeal. This was to ensure fly midguts were relatively free of blood, which improves the accuracy of scoring midguts for trypanosome infection. For scoring infection status, midguts were shredded into 100 µL of saline on a glass slide. The infection status was determined by searching 10 random fields by dark field microscopy (125× magnification) for motile trypanosomes.

### Estimation of bloodmeal size

To determine the average bloodmeal size ingested by *G. m. morsitans*, nine male and nine female flies of each age group were caged individually and weighed before and after membrane feeding. Immediately after feeding, the bottom of each cage was sealed with parafilm to prevent the loss of any droplets due to diuresis. Flies were observed throughout feeding and, upon fly detachment from membrane (full engorgement), the fly weight (mg) was immediately recorded. This procedure was repeated for 24 h.p.e. and 48 h.p.e. flies.

### One-dimensional gel electrophoresis and immunoblotting

A group of newly emerged flies was collected during a four hour time period and flies were immediately separated into two groups. The first group received a blood meal at 24 h p.e. and the second group remained unfed. Every 24 hours, tsetse midguts were dissected in PBS, collected into 1.5 mL microcentrifuge tubes (in pools of five) and the tubes and contents were snap-frozen in liquid nitrogen. Three timepoints (24 h, 48 h and 72 h) were collected from each group to compare differences in Pro2 expression. Midgut samples from fed flies were first collected 24 hours after the bloodmeal, and thus represent flies that are aged 48 h.p.e. Tissues were resuspended in 20 µL of 1× Laemmli buffer [23], heat denatured for 10 minutes and briefly centrifuged to pellet the insoluble fraction. Samples were stored at −80°C until required. Each denatured sample (20 µL = half of a midgut equivalent) was electrophoresed using 10% polyacrylamide gels and processed as described by [24]. A 10 kDa molecular marker (PageRuler™ Unstained Protein Ladder, Fermentas Life Sciences #SM0661), was run on each gel for accuracy in determining the molecular mass of immunoreactive protein bands.

The anti-proventriculin 2 (anti-Pro2) mouse monoclonal antibody (mAb 4A2) was a gift from Professor Terry Pearson (University of Victoria, British Columbia, Canada). It is a mouse monoclonal (isotype IgM) that recognizes *Glossina sp.* Pro2 (GenBank: AAN52277.1) and was initially derived from mice injected with sonicated midgut material (teneral *G. m. morsitans*) suspended in PBS [25]. Immunoblotting using Hybond™- P polyvinylidene difluoride (PVDF) transfer membrane (Amersham Biosciences, Amersham, UK) was performed as previously described [26]. In brief, a 1∶20 dilution of anti-Pro2 mouse monoclonal antibody in 5% skim milk in PBS (w/v) was used. The secondary (detecting) antibody was a 1∶50,000 dilution of horseradish peroxidase conjugated goat anti-mouse IgG/IgM (H+L) (Caltag Laboratories, South San Francisco, CA). The western blot was developed with SuperSignal Dura chemiluminescence substrate (Pierce Chemical Company, Rochford, IL) and Kodak Biomax MR film (Eastman Kodak Company, Rochester, NY) was used to detect chemiluminescence. After development of the autoluminograms, proteins were stained on the PVDF membrane with 0.2% (w/v) nigrosine in PBS. The exposed film was superimposed on the stained PVDF membrane to reveal the precise location of the immunoreactive protein bands in relationship to the entire protein profile and to ensure equivalent protein loading in each lane.

### Statistical analysis

Statistical analysis was performed using SPSS16 (SPSS Inc., Chicago, Illinois). Fisher's exact test was performed to determine if significant differences in trypanosome infection rates were present between experimental groups. ANOVA was used for analysis of bloodmeal size.

## Results

### Midgut susceptibility to parasite infection changes with age of newly emerged flies

**Figure 1:**
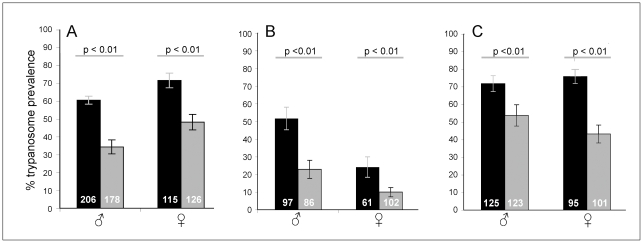
The relationship between trypanosome prevalence in the midgut and the age of the teneral fly. Male and female tsetse aged 24 h.p.e (black bars) and 48 h.p.e (grey bars) were offered a bloodmeal containing trypanosome parasites. Panel A shows the midgut infection prevalence of teneral *G. m. morsitans* infected upon the first bloodmeal with *T. b. brucei* TSW196 BSF; Panel B represents teneral *G. p. palpalis* infected with *T. b. brucei* TSW196 BSF; Panel C corresponds to teneral *G. m. morsitans* infected with *T. congolense* 1/148 BSF. The white numbers within the bars indicate the total number of flies dissected from three replicate experiments.

To determine if there are differences in susceptibility between “young” (24 h.p.e.) and “old” (48 h.p.e.) teneral flies, replicate experiments were conducted on both male and female *G. m. morsitans* infected with *T. b. brucei* TSW196 BSF trypanosomes. Figure 1, Panel A illustrates the variation in infection rates that exists between young and old *G. m. morsitans* teneral flies of both sexes. The graph includes the collective data from five male and three female replicate experiments (total fly numbers indicated in white). The difference in midgut infection rates between the two time points was statistically significant for both sexes (Fisher's exact test: male, ρ<0.01; female, ρ<0.01). The reproducibility of all the replicates remained remarkably consistent as evidenced by the relatively narrow standard error of the mean (S.E.M.) value for each group in Panel A.

### Age-related refractoriness is conserved in different vector/parasite pairings

To determine if the teneral phenomenon was specific to our laboratory vector-parasite combination of *G. m. morsitans* and *T. b. brucei* TSW196 BSF, we undertook further investigations using another tsetse species, *G. p. palpalis* infected with *T. b. brucei* TSW196 BSF trypanosomes. Figure 1, Panel B shows the differences in trypanosome susceptibility between “young” (24 h.p.e.) and “old” (48 h.p.e.) teneral flies of different sexes. Statistically significant differences in infection rates were found in both male and female flies (Fisher's exact test: male, ρ<0.01; female, ρ<0.01).

The same age-related trend in susceptibility was also observed when both male and female *G. m. morsitans* were infected with another salivarian trypanosome species, *T. congolense* 1/148 BSF (Figure 1, Panel C). Again, the difference in infection rate between the young and old flies was deemed statistically significant (Fisher's exact test: male, ρ<0.01; female, ρ<0.01).

### Size of the ingested bloodmeal does not explain the teneral phenomenon

**Figure 2:**
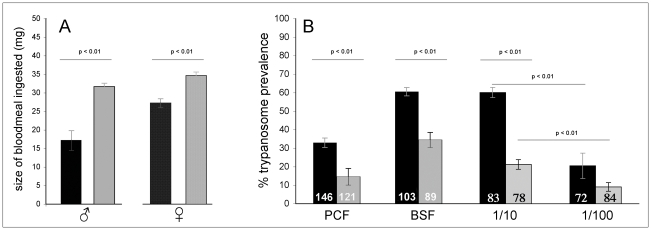
Comparison of average bloodmeal volumes and midgut infection rates in teneral *G. m. morsitans*. Male and female flies were aged 24 h.p.e (black bars) or 48 h.p.e. (grey bars) at the time of the infective bloodmeal. Panel A shows the significant differences in volume (mg) of a first bloodmeal ingested by young and old male and female flies. Panel B shows the variability of midgut infection rates in young and old male flies fed PCF or different concentrations of BSF *T. b. brucei* TSW196. Parasite (BSF) concentrations in the bloodmeal were 5×10^4^ trypanosomes/mL (1/10) and 5×10^3^ trypanosomes/mL (1/100). The numbers within the bars represent the number of flies scored from three replicate experiments.

To ensure that the difference in bloodmeal size and the number of parasites ingested did not contribute to elevated infection rates in young tenerals, we measured the average volume of blood ingested by male and female *G. m. morsitans* aged 24 h.p.e. and 48 h.p.e. It was observed that young teneral flies of both sexes take significantly smaller bloodmeals than old teneral flies (male: F = 29.52, df = 1, ρ<0.01; female: F = 16.53, df = 1, ρ<0.01) as shown in Figure 2, Panel A. A reasonable prediction based on bloodmeal size and the original trypanosome concentration is that younger flies ingested 8.6×10^3^ trypanosomes (males) or 1.3×10^4^ trypanosomes (females) on average compared to 1.6×10^4^ (males) or 1.7×10^4^ (females) trypanosomes ingested by older flies. Yet, despite the increased parasite load, older flies are more resistant to infection (Figure 1). So, within the bounds of the parasite numbers ingested in this experiment, the trypanosome load does not contribute to the teneral phenomenon. This is in accordance with the data shown in Figure 2, Panel B, which demonstrates that the infectious dose must be diluted by at least 10 fold before any effects are seen on midgut infection prevalence in young and old male *G. m. morsitans* flies (Fisher's exact test: p<0.01 (24 h.p.e.); p<0.01 (48 h.p.e.)).

### The life cycle stage of the parasite does not affect the teneral phenotype

To determine if this age-related teneral phenotype only appeared when feeding BSF trypanosomes, we fed tsetse flies with *T. b. brucei* TSW196 PCF trypanosomes. To maintain experimental parameters as uniform as possible, log phase PCF were adjusted to a concentration of 5×10^5^ trypanosomes/mL of blood, the same concentration used for feeding of BSF trypanosomes. Figure 2, Panel B shows the infection rates for male *G. m. morsitans* fed PCF trypanosomes. Overall, infection with PCF trypanosomes resulted in lower midgut infection prevalences than infection with BSF trypanosomes. This may be attributed to the fact that PCF trypanosomes were added to fresh horse blood that presumably still had residual complement activity (PCF are known to be sensitive to vertebrate complement [27]). However, despite this observation, the age related effect on the teneral phenomenon is still seen in flies fed a first bloodmeal containing PCF trypanosomes. Younger teneral flies exhibited a higher susceptibility than older teneral flies to trypanosome infection, which again was statistically significant (Fisher's exact test: ρ<0.01).

### Teneral infection rates are influenced by bloodmeal parasitaemia

To determine if bloodmeal parasitaemia has an effect upon the teneral phenomenon, flies were fed parasite-infected bloodmeals that contained 10 and 100 fold fewer trypanosomes (*i.e.* 5×10^4^ trypanosomes/mL and 5×10^3^ trypanosomes/mL) than the normal 5×10^5^ trypanosomes/mL. Both infectious doses still resulted in higher infection rates in young compared to old teneral flies (Figure 2, Panel B) and the differences were statistically significant (Fisher's exact test: ρ<0.01). However, it should be noted as it has been by others [28], that there is an overall decline in the prevalence of midgut infections as the number of parasites ingested declines. It seems the threshold for this decrease in infection prevalence falls between 5×10^4^ and 5×10^3^ as it is not seen when parasite numbers decline from 5×10^5^ trypanosomes/mL to 5×10^4^ trypanosomes/mL (compare Figure 1, Panel A with Figure 2, Panel B).

### Linear relationship between prevalence of infection and hours post eclosion

**Figure 3:**
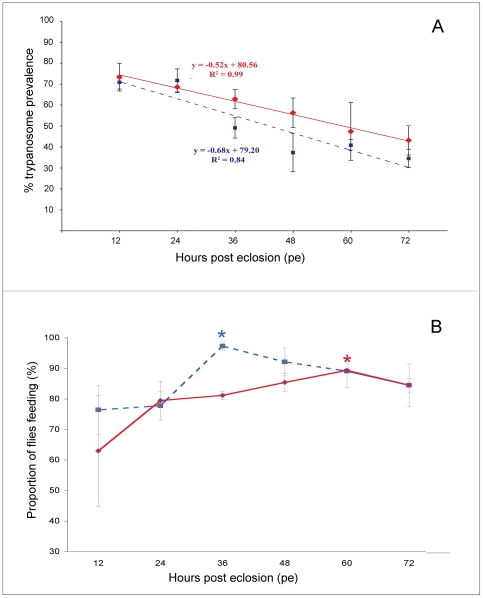
The age-prevalence of teneral *G. m. morsitans* midgut infections. Male (broken blue line) and female (solid red line) infection data are depicted. Flies were collected every 12 h over three days and infected with a first bloodmeal containing *T. b. brucei* TSW196 BSF. Panel A graphs the percentage of immature midgut infections (y-axis) versus the post emergence age of teneral tsetse (x-axis). Regression analyses are shown as equations. Panel B compares the percentage of flies that accepted the first bloodmeal (y-axis) against fly age p.e. (x-axis). Asterisks mark the age at which male (blue) and female (red) have the highest probabilities of feeding post emergence. Flies were collected from three independent experiments.

Groups of *G. m. morsitans* male and female flies infected at twelve hour intervals with *T. b. brucei* TSW196 BSF show a linear decline in prevalence of infection up to the conclusion of our experiment at 72 h.p.e. (Figure 3, Panel A). A regression analysis was performed for male flies (blue broken line) (y = −0.68x+79.20, R^2^ = 0.84) and female flies (red solid line) (y = −0.52x+80.56, R^2^ = 0.99). Statistical analysis of male and female data sets indicated that there is a statistically significant difference in midgut infection rates based on the age of the fly (Fisher's exact test: ρ<0.001 (male); p<0.001 (female). There was no significant difference in mortality rates noted between the 0–72 h.p.e. aged groups and the average mortality observed was less than 20% for both males and females (data not shown). The proportion of flies that willingly fed at various times p.e. is shown in Figure 3, Panel B.

### Does a correlation between midgut infection prevalence and length of the peritrophic matrix (PM) exist?

**Figure 4:**
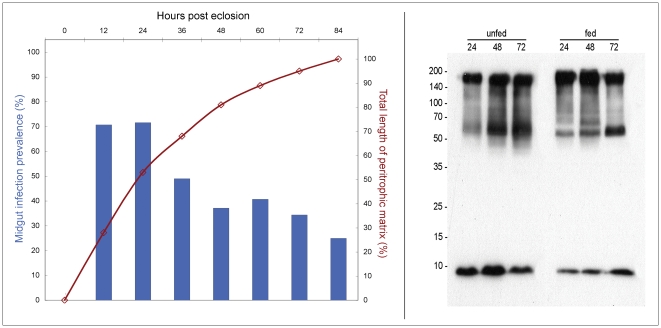
The parasite prevalence in infected teneral male *G. m. morsitans* compared to the mean length of the PM (graph) and an immunoblot of tsetse proventriculin 2 expression (Pro2). The graph depicts midgut infection data (derived from Figure 3) plotted on the y-axis (left) and the mean length of the PM (converted to a percentage of the fully formed length) derived from Lehane and Msangi [30] on the y′-axis (right). The fly age (h.p.e.) is represented on the x-axis. The red line indicates the formation of the PM over an 84 hour time course. The blue bars show the gradual decline in trypanosome prevalence with fly age. The midgut infection rates are inversely proportional to the length of type II PM secreted by the proventriculus. The Western blot illustrates the differential expression of midgut protein Pro2 in teneral unfed flies (aged 24 h.p.e., 48 h.p.e. or 72 h.p.e) and fed flies (24, 48 or 72 hours post bloodmeal). Fed flies received a bloodmeal at 24 h.p.e. and were dissected at 24 hour intervals. Half of a midgut equivalent (from a pool of five) was loaded per lane and detected with mAb 4A2, a monoclonal antibody that recognizes tsetse Pro2.

The PM is continually secreted by the proventriculus throughout the life of the fly. However, in teneral flies, the length of the PM dramatically increases as the fly ages. Since the PM presents a physiological barrier to parasite invasion [29] it was hypothesized that a correlation might exist between the teneral phenomenon (susceptibility) and PM maturity. Lehane and Msangi [30] measured the length of the emergent teneral PM (in male *G. m. morsitans*) over a time course of 84 h.p.e. The length of the growing PM was converted into a percentage of the final length (∼43 mm) and then plotted against fly age post-eclosion. Newly emerged flies (<24 h.p.e.) have a partially formed/immature PM. Using a graph (Figure 4), where matrix length was plotted as a function of time, the infection prevalence data from male, *G. m. morsitans* infected with *T. b. brucei* TSW196 (data summarized in Figure 3, Panel A) was superimposed. A distinct correlation between the two emerges; at 36 h.p.e., the PM reaches half of its mature length and the midgut infection rate is just under 50%. Only 12 hours later, the infection rate falls to ∼35% and the PM reaches greater than 80% its final length. Decreased midgut infection rates are inversely proportional to the mature length of type II PM. Whether this is a function of the physiological immaturity of the midgut remains to be determined.

The graph in Figure 4 illustrates how the length of the PM differs between teneral flies of different ages. To further validate this observation with molecular techniques and to show that indeed differential protein expression exists between midguts isolated from young and old unfed teneral flies and age-matched fed flies, immunoblotting was performed using the anti-Pro2 monoclonal antibody, mAb 4A2 (Figure 4, Western blot). Pro2 is a protein localized to the tsetse PM. Similar to the peritrophin-15 family, Pro2 (NCBI Accession number AAN52277.1) is likely synthesized and secreted by the proventriculus [31]. The anti-Pro2 monoclonal antibody is represented by a distinct banding pattern on tsetse midgut tissue with an upper protein band at ∼170 kDa, a large smeary band (likely due to post-translational protein modifications) between 60 and 170 kDa and a small ∼8 kDa band. A higher Pro2 abundance was observed in an unfed 48 h.p.e. fly compared to either a 24 h.p.e. or 72 h.p.e. fly. Fed flies had a slightly higher abundance of the ∼170 kDa band and a lower abundance of the ∼8 kDa lower band in comparison to age-matched unfed flies (48 h.p.e. unfed = 24 h fed, 72 h.p.e. unfed = 48 h fed). Therefore, the protein expression patterns of Pro2 in midguts isolated from newly emerged fed and unfed flies change in response to both fly maturation and feeding. This further supports the contention that the molecular composition of the teneral midgut varies between younger and older flies, reinforcing our suggestion that it is an error to group all newly emerged flies together as one physiological state (teneral).

## Discussion

Cost considerations and logistical difficulties with tsetse colonies usually mean there is a constraint on fly numbers available to researchers. Consequently, when large numbers of teneral flies are required to enable a comprehensive experiment design, it is common practice to expand fly collection times and to treat all flies emerging over a few days as one large, homogeneous “unfed” group. Clearly, from our data, it can be seen that this can severely distort the outcome of experiments involving trypanosomes. For example, if the collection of teneral flies proceeds over three days, then the spread of susceptibility built into the experiment at the outset will range from >70% susceptibility in 12 hour old flies to <50% in flies 72 hours old. If collection includes even older teneral flies, then an increase in susceptibility is seen as the effect of starvation becomes apparent [7]. This will introduce large fluctuations in error between replicate experiments. It is clear from others' work that the absolute degree of susceptibility observed will also vary with the fly and trypanosome strain used [32]. The teneral phenomenon is probably widespread, as we have demonstrated it here using two tsetse species and two trypanosome species (Figure 1). Knowing this, cohorts of teneral flies of the same age p.e. must be used in all replicates and preferably, the teneral age should be recorded in terms of hours after emergence rather than in days.

In the laboratory, susceptibility of flies to *T. congolense and T. brucei* infections typically falls rapidly from as high as 70% to 10% and less following the third bloodmeal [3], [4], [5], [6], [7]. This refractory phenotype observed in flies that have taken multiple bloodmeals is sustained throughout the lifespan of the fly, although factors such as starvation [7], [33], [34] and environmental stressors [1] may partially reverse this trend. In the field, while *T. congolense* infections can be acquired at any age [35], [36], [37], there is still a decline in susceptibility with fly age. This is either due to age-associated decrease in susceptibility or an increasing population of completely refractory flies [38]. The situation for *T. brucei* infections is unknown because of the low levels of fly infection seen in the field and the subsequent lack of suitable data for analysis. While the teneral phenomenon may not have the profound effect in the wild that it has in the laboratory, it is probably still an important epidemiological factor. The question then is how soon after emergence do flies feed in the field? Wild teneral *G. m. morsitans* are not usually active during the first two days p.e. [39]. However, we might expect that under the stressful environmental conditions of a hot, dry season when flies are concentrated at water holes, they may be more likely to feed soon after emergence.

In contrast to previous reports [10], [40], [41], [42], some of our results show *G. m. morsitans* teneral female flies (both young and old) were more susceptible to parasite infection than their male counterparts. This increased female susceptibility has also been reported by others [12]. However, this sex bias was not repeated when female *G. p. palpalis* were infected with the same parasite strain (*T. b. brucei* TSW196). Considering our own and previously published data, we conclude that sex based bias in vector competence changes with different vector-parasite combinations and that the underlying mechanisms causing shifts in the bias are not known.

We investigated if the number of trypanosomes (or bloodmeal volumes) ingested by young and old teneral *G. m. morsitans* of both sexes influenced prevalence of infection with *T. b. brucei* BSF trypanosomes. For both males and females, the size of the bloodmeal taken by younger flies is significantly smaller than that taken by older flies, so that consequently fewer trypanosomes are ingested. However, midgut infection rates were higher in younger flies despite the fact that male 48 h.p.e. flies theoretically ingest twice as many trypanosomes than a 24 h.p.e. fly. The same observation was true for female flies, but to a lesser degree. So, within the limits of the numbers of trypanosomes ingested in those experiments, neither the size of the bloodmeal nor the number of trypanosomes ingested is the determining factor in the prevalence levels observed. However, if trypanosome numbers are reduced to 5×10^3^ trypanosomes/mL, then trypanosome numbers clearly influence infection outcome in the fly (Figure 2, Panel B). The drop in infection prevalence seen in the fly when parasite numbers are reduced to 5×10^3^ trypanosomes/mL (i.e. 150 trypanosomes/fly/30 µl bloodmeal) is large enough (Figure 2, Panel B) that it will mask any teneral effect in a poorly designed experiment. For example, 48 h.p.e. flies ingesting blood that contains a parasite concentration of 5×10^3^ trypanosomes/mL had a midgut infection prevalence of only 8.85%. This is equivalent to the natural refractory phenotype reported in older *G. m. morsitans,* which have fed twice before [6]. Therefore, during experimental planning, it is important to consider that the number of trypanosomes given in infected bloodmeals remains above the threshold mentioned above. It seems possible that this threshold may vary with fly/trypanosome combination and will thus require some preliminary experiments if new combinations are used. The parasitaemia of the host may contribute to the probability of infection under natural conditions, but possibly only if the number of parasites ingested is either extremely high or very low [10], [43] and/or depending on the development phase of the parasite within the host [44], [45].

In several publications tsetse have been infected by feeding them with PCF trypanosomes instead of BSF trypanosomes [14], [15]. As only the stumpy BSF trypanosomes are pre-adapted for survival in the midgut [46], comparing the prevalence of infection achieved by feeding different parasite lifecycle stages was of interest. Feeding male teneral *G. m. morsitans* with bloodmeals spiked with either PCF or BSF trypanosomes of the same strain (*T. b. brucei* TSW196) resulted again in a clear distinction between the parasite-susceptibility of young and old flies. It is therefore doubtful that the trypanosome lifecycle form is responsible for this age-related teneral phenomenon. Of note, the PCF trypanosomes were less able to establish midgut infections despite being pre-adapted for insect midgut conditions. This is likely to be because procyclic trypanosomes are complement sensitive [27], [47]. When PCF trypanosomes are diluted in PBS-washed red blood cells reconstituted with heat-inactivated serum, midgut infection rates will increase significantly in number [48]. Also if the researcher needs maximum numbers of infected flies, then infection with BSF rather than PCF trypanosomes is recommended (Figure 2, Panel B).

The data, derived from this compilation of experiments, indicate that there is a significant negative correlation (ρ<0.0005) between age (h.p.e.) and the prevalence of parasite infection in a fly population (Figure 3). Others have found that there is no change in infection over the first two days p.e. and then said that starvation effects at 3 and 4 days p.e. lead to increased prevalence of infection of flies [7]. The reason for these differences between the two data sets is not known.

The molecular status of the fly midgut differs between younger and older teneral flies. As the teneral fly is developing, many physiological changes are occurring, including the molecular environment of the midgut. In this paper, we demonstrate a distinct correlation between length and physiological maturity of the PM (by analysis of Pro2 protein that is localized to the tsetse PM) and fly susceptibility to trypanosome midgut infection (see Figure 4). In addition to investigating PM maturity, we examined the teneral midgut for the presence of the larval meal and midgut-associated symbionts. Although Rio *et al.* [49] noted a dramatic increase in symbiont density p.e., the abundance of GroEL (a symbiont-associated heat shock protein (Hsp60)) significantly decreases as the teneral fly ages. Rapid disappearance of milk gland protein (MGP), a constituent of the larval meal remaining in the midgut upon eclosion, was likewise observed (see Figure S1, Text S1). The elevated presence of MGP and of the symbiont-specific GroEL protein corresponds well with the increased infection prevalence data in young emergent flies. Although there are several co-correlations presented here, the key molecular mechanism(s) underpinning the teneral phenomenon remain unresolved.

Overall, our recommendation to optimize numbers of flies with midgut infections would be to feed male flies at 36 h.p.e. (∼55% infection) and females at 60 h.p.e. (∼50% infection). While feeding earlier may give higher prevalences of infection, flies that are less than 12 hours old do not readily feed and are fragile and thus difficult to handle without causing undue stress to the insect [10]. However, we feel that it will be necessary to confirm these findings again when using different fly trypanosome combinations other than *G. m. morsitans* infected with *T. b. brucei* TSW196 BSF. By adopting the practice of defining ‘teneral’ in terms of “age post eclosion” (instead of simply “unfed”), researchers working on vector competence in *Glossina* will achieve superior experimental reproducibility and accuracy as well as optimizing the numbers of infected flies available for further experiments.

## Supporting Information

Figure S1 **Immunoblot analysis of heat shock protein (Hsp60) and tsetse milk gland protein (MGP) in teneral male** ***G. m. morsitans*** **midguts.** One midgut equivalent was loaded per lane of a 10% polyacrylamide gel. The PVDF membrane was stained with nigrosine (upper purple membrane) to ensure equal protein loading per lane. Lane 1: 0–4 h.p.e., Lane 2: 4–8 h.p.e.; Lane 3: 8–12 h.p.e.; Lane 4: 12–16 h.p.e.; Lane 5: 20–24 h.p.e.; Lane 6: 44–48 h.p.e.; Lane L: molecular mass ladder. Hsp60 = 60 kDa; MGP = 23 kDa.
(TIF)

Text S1 **Supplementary materials and methods.**
(DOC)
