# Supplementary figures and images for: Post Eclosion Age Predicts the Prevalence of Midgut Trypanosome Infections in Glossina
Source: PLoS One. 2011 Nov 8;6(11):e26984. doi: 10.1371/journal.pone.0026984 (PMC3210762; doi:10.1371/journal.pone.0026984)

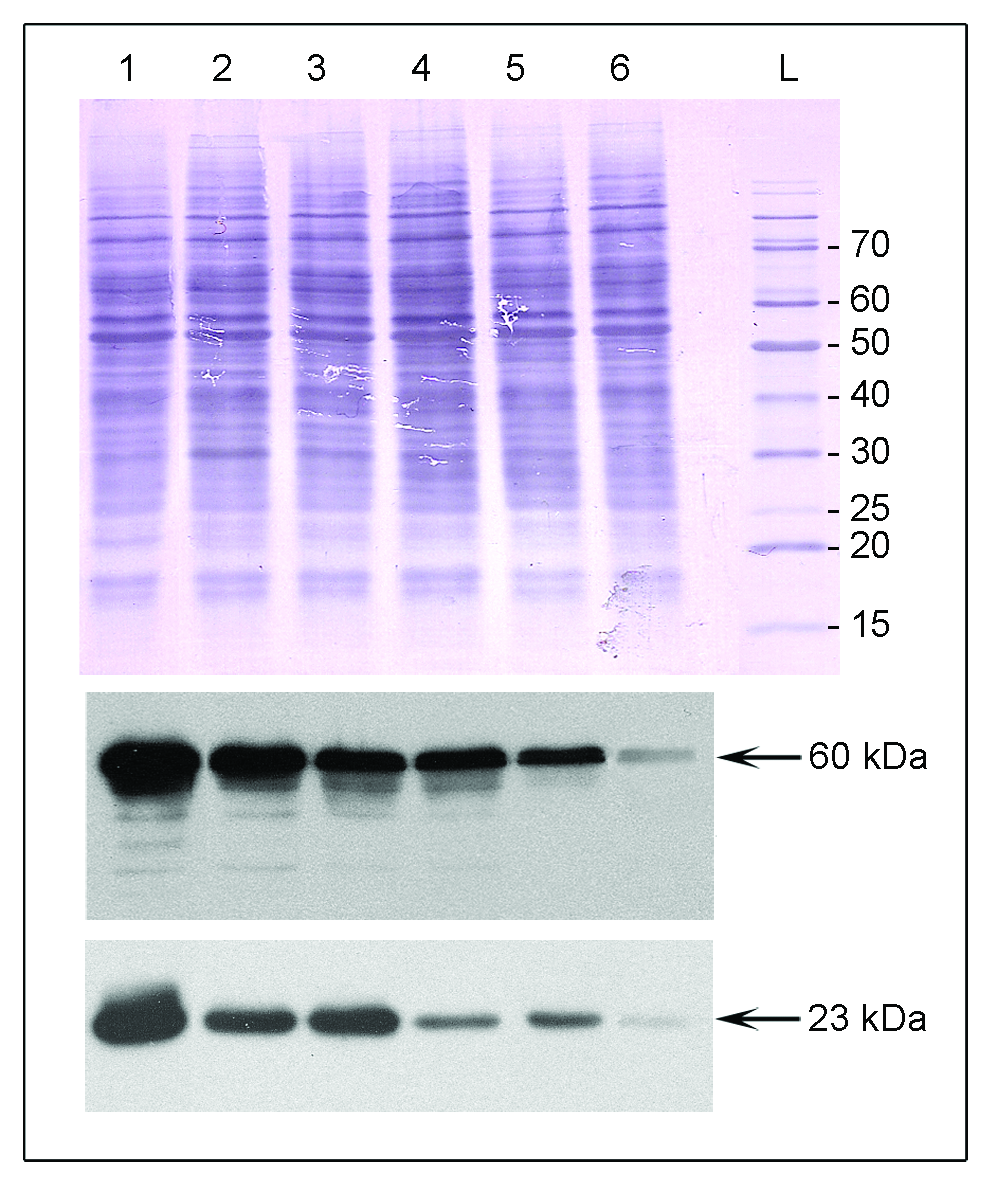

Supplement: Figure S1 — Immunoblot analysis of heat shock protein (Hsp60) and tsetse milk gland protein (MGP) in teneral male G. m. morsitans midguts. One midgut equivalent was loaded per lane of a 10% polyacrylamide gel. The PVDF membrane was stained with nigrosine (upper purple membrane) to ensure equal protein loading per lane. Lane 1: 0–4 h.p.e., Lane 2: 4–8 h.p.e.; Lane 3: 8–12 h.p.e.; Lane 4: 12–16 h.p.e.; Lane 5: 20–24 h.p.e.; Lane 6: 44–48 h.p.e.; Lane L: molecular mass ladder. Hsp60 = 60 kDa; MGP = 23 kDa. (TIF) [file pone.0026984.s001.tif]
