# Supplementary material for: Post Eclosion Age Predicts the Prevalence of Midgut Trypanosome Infections in Glossina
Source: PLoS One. 2011 Nov 8;6(11):e26984. doi: 10.1371/journal.pone.0026984 (PMC3210762; doi:10.1371/journal.pone.0026984)
Supplement: Text S1 — Supplementary materials and methods. (DOC) [file pone.0026984.s002.doc]

Supplementary Materials and Methods

**Sources of monoclonal antibody and polyclonal antiserum**

**Rabbit Anti-GmmMGP polyclonal**

The polyclonal antiserum that recognizes *G. m. morsitans* milk gland protein was a gift from Dr. Geoff Attardo (Yale University School of Medicine, New Haven, Connecticut, USA). The tsetse milk gland sequence was bacterially expressed in *E. coli* BL21 cells as inclusion bodies. These bodies were subsequently purified and separated by SDS-PAGE. The protein band corresponding to the purified product was used to generate commercially produced rabbit antiserum [1].

**Anti-symbiont mouse monoclonal**

The anti-GroEL (mAb 1H1) antibody was a gift from Prof. Terry Pearson (University of Victoria, British Columbia, Canada). mAb 1H1 is a mouse monoclonal (isotype IgG2a) that cross-reacts with all Enterobacteriaceae homologues of GroEL and was originally derived from mice injected with heat-killed secondary tsetse symbiont, *Sodalis glossinidius*.

**Western blot analysis of *G. m. morsitans* milk gland protein and symbiont protein**

Western blot analysis was performed as described in the main Materials Section. Tsetse midguts (pool of two flies) were collected at four hour intervals and solubilized in Laemmli buffer. One midgut equivalent was loaded per lane of a 10% polyacrylamide gel. The proteins were blotted and detected with polyclonal rabbit anti-tsetse milk gland protein, which specifically binds to a 23 kDa molecule (lower panel). The same membrane was stripped using Restore^TM^ PLUS Western Blot Stripping Buffer (Thermo Scientific, Rockford, IL) and reprobed with a mouse anti-GroEL (mAb 1H1) monoclonal antibody which detected a 60 kDa heat shock protein (Hsp60) expressed by both *W. glossinidia* and *S. glossinidius* (middle panel). PVDF membranes were stained with nigrosine (upper purple membrane) to ensure equal protein loading per lane.

**References to Supplementary Material**

1. Attardo GM, Guz N, Strickler-Dinglasan P, Aksoy S (2006) Molecular aspects of viviparous reproductive biology of the tsetse fly (*Glossina morsitans morsitans*): regulation of yolk and milk gland protein synthesis. J Insect Physiol 52: 1128–1136.
